# Experiences of Parents and Caregivers of Children Who Underwent Gastrostomy Tube Insertion

**DOI:** 10.1177/23743735241272225

**Published:** 2024-08-21

**Authors:** Ciara Kinsella, Aisling Dunphy, Siobhan McCormack, Charlotte Wilson, Annemarie E Bennett

**Affiliations:** 1 Children's Health Ireland at Tallaght, Tallaght University Hospital, Dublin, Ireland; 2 Department of Clinical Medicine, Trinity Centre for Health Sciences, St James’ Healthcare Campus, Dublin, Ireland

**Keywords:** gastrostomy, gastrostomy insertion, parental experience, caregiver experience, pediatrics, tube feeding

## Abstract

Gastrostomy feeding is a route of enteral nutrition for children with feeding difficulties. This study investigated caregiver experiences of the transition to gastrostomy feeding. A survey was administered to caregivers of children <18 years in a major pediatric center in Ireland. Experiences of decision-making, support, and adjusting to tube feeding were examined. Seventy-six caregivers participated. Median satisfaction with the information provided by the hospital was high. Almost half (48%) spoke to another caregiver of a child with a gastrostomy prior to their own child's gastrostomy insertion and most (88%) felt reassured by this. Concerns following insertion included managing the tube and their child's oral intake and feelings about the tube. The oral intake of 83% of children who had some intake prior to gastrostomy insertion did not change or increased following insertion. Most (89%) would make the same decision to insert the tube. Feelings associated with the transition included relief and stress. Gastrostomy tube insertion presents logistical and psychosocial challenges for caregivers. Peer support from other caregivers may alleviate some of these challenges.

## Introduction

Enteral nutrition support involves the provision of nutrients to the gastrointestinal tract via specialized feeding tubes.^1^ A gastrostomy is a tube that enters the stomach via a stoma in the abdominal wall and is a feeding route suited to the long-term provision of enteral nutrition.^2^ A gastrostomy tube is most commonly placed endoscopically but may also be placed surgically or radiologically.^1^ Gastrostomy feeds can be provided continuously via a pump and/or as boluses.^3^ A feeding regimen should meet a child's nutritional requirements and be tailored to their day-to-day lifestyle. Gastrostomy feeding is indicated in children who have a functioning digestive system but who cannot safely swallow; cannot meet nutritional requirements by oral intake alone; and/or, require a gastrostomy tube solely for the provision of fluids and medicines.^4^

There are benefits and risks to gastrostomy feeding. Benefits include weight gain, increased subcutaneous fat stores, decreased duration of mealtimes, decreased risk of respiratory illness, and improved quality of life for children and carers.^5–9^ Risks of gastrostomy feeding principally include those associated with the surgical placement of the gastrostomy. Minor complications that can occur at a later stage post-insertion include stoma leakage, cellulitis, or the formation of granulation tissue around the gastrostomy site.^10,11^

The decision to place a gastrostomy tube is often complex^12^ and can significantly impact on a child, their caregivers, and families in physical, emotional, and relational respects.^13,14^ Parents may be reluctant to consent to gastrostomy insertion as they may view this as a parental failure in their duty to feed their child.^13^ Parental beliefs on the meaning of feeding have been described as “more than a means to a good nutritional state.”^13^ While oral feeding is often challenging, by placing a gastrostomy tube, parents may feel that they are denying their child the social and sensory pleasure of mealtimes.^15^ Parents may also feel that the initiation of gastrostomy feeding represents the start of a decline in their child's condition, a loss of normality, and a visible symbol of disability.^14,16^ Parents may be conflicted between the possible negative impacts of gastrostomy feeding for their child and the potentially improved quality of life for them, as caregivers.^13^ Significant factors influencing their decision may include the birth of another child, a video fluoroscopy result, or their child developing an acute critical illness.^13^

Research has demonstrated that the initiation of gastrostomy feeding is accompanied by positive and challenging considerations that impact on relationships within a family, between family and healthcare providers, and between a family and wider society.^14^ Given the potentially significant impact of gastrostomy feeding, these experiences should be further examined to increase the provision of care that is not only patient-centered but family-centered.

Limited research exists on how caregivers perceive the support provided by health professionals during the process of gastrostomy insertion. While evidence exists to encourage peer support between caregivers of children with complex neurodisabilities,^17^ the impact of support between caregivers of children requiring a gastrostomy insertion has yet to be studied. Exploring caregiver perspectives on the decision-making process and on life after gastrostomy insertion would improve health professionals’ understanding of how to optimize this experience for caregivers and ensure the best possible outcomes for each child in need of this form of nutritional support.

## Methods

A cross-sectional self-administered online survey was conducted in March 2023. Participants were recruited from inpatient and outpatient departments in Children's Health Ireland (CHI) at Tallaght, CHI at Crumlin, and CHI at Temple Street. Inclusion criteria included parents or caregivers of children who were <18 years old, attending a consultant at CHI at Tallaght, CHI at Crumlin, or CHI at Temple Street, and/or who had a gastrostomy inserted with CHI at Tallaght, CHI at Crumlin, or CHI at Temple Street. Children who received some/all their nutrition, fluids, and/or medications via a standard gastrostomy, gastrostomy with jejunal extension, or a gastrostomy button, were included. Children without gastrostomy tubes or who had gastrostomy tubes inserted outside of CHI were excluded, due to the specific scope of this study.

Participants were invited to complete a 20-item Qualtrics© online survey. Informed consent was obtained at the beginning of the survey. Closed-ended questions and sliding scales assessed caregiver understanding of why the gastrostomy tube was inserted, their involvement in the decision, and their view of the timing of the insertion. Caregiver understanding of the reason for gastrostomy tube insertion and satisfaction with the information provided by the medical team were rated on 10-point Likert scales, where 0-3 = poor, 4-6 = good, 7-8 = very good, and 9-10 = excellent. The perceived impact of speaking with other caregivers who had a child with a gastrostomy and their views on their child's oral intake and the post tube insertion period were also examined. The survey was designed for this study and was not assessed for reliability. Face validity of the questions was determined through consultation with the research team. No other forms of validity were determined.

The survey was piloted in CHI at Tallaght. Due to time constraints for fieldwork, only face validity was assessed. Four caregivers were asked for feedback on the accessibility of the survey, time taken to complete it, and phrasing of questions. No questions were added or removed, but alterations to the phrasing of questions and additional possible answers to questions were made.

The survey was administered across 3 CHI locations (Tallaght, Crumlin, and Temple Street). Parents or caregivers of children attending outpatient dietetics appointments, in-person or virtually, were invited to participate using convenience sampling. Eligible parents of children who were inpatients were also invited to participate using convenience sampling. A poster promoting the survey and containing a QR code to make the survey more accessible was provided to eligible participants who expressed an interest in the study. Paper copies of the survey were also provided for participants with no access to a smartphone. Data from paper surveys were inputted manually into the Statistical Package for Social Sciences (SPSS).

Data analyses were conducted using the SPSS for Windows 11, version 28.0 (IBM Corporation). Descriptive data were presented using percentages and frequencies. Normally distributed data were numerically summarized using the mean and standard deviation. Non-normally distributed data were summarized numerically using the median and interquartile range (IQR). Associations between normally distributed data and categorical data were explored using an analysis of variance test. Bivariate correlations assessed the direction of a relationship between 2 variables. The Spearman correlation coefficient (rho) assessed the relationship between non-normally distributed variables. Statistical significance was taken at *P *< .05.

Responses to the open-ended question were grouped into positive, negative, and neutral responses. Participants could provide up to 3 phrases/words describing the experience. Positive comments were defined as those indicating satisfaction with the process of gastrostomy insertion and/or experiencing the benefits of the gastrostomy insertion,^18^ for example, “happy,” “relief,” “better intake,” and “discreet.” Negative responses were those reflecting a feeling of distress with, or experiencing a complication during, the process of gastrostomy insertion, for example, “fear,” “frustration,” “granulation,” and “infection.”^18^ Responses not expressing emotion strong enough to fit either definition were categorized as neutral, such as “necessary.”^18^ A word cloud (www.worditout.com) was created to present the most common words submitted, with more common words appearing larger in the word cloud, relative to less common words.

## Results

### Demographic Characteristics

Seventy-six responses eligible for analysis were received (Table 1). Ten (13.2%) were partially complete and 66 (86.8%) were fully complete. Most (85.5%, n = 65) participants were mothers, 9.2% (n = 7) were fathers, 1.3% (n = 1) were siblings, and 3.9% (n = 3) were other caregivers. The median (IQR) age at gastrostomy insertion was 13.5 (8.5-36) months. Over half (56.6%, n = 43) had the gastrostomy tube inserted for >3 years, while 21.0% (n = 16) had the tube inserted for <6 months. Over three-quarters (76.8%, n = 53) of children had a nasogastric or nasojejunal tube inserted prior to the insertion of the gastrostomy tube.

**Table 1.** Characteristics of Primary Caregivers Who Responded to a Survey on their Experience of their Child Undergoing Gastrostomy Insertion.

	Median	IQR
Median age at gastrostomy insertion (months)	13.5	8.5, 36
	n	%
Relationship to child (n = 76)		
Mother	65	85.5
Father	7	9.2
Sibling	1	1.3
Other	3	4.0
Duration since gastrostomy insertion (n = 76)		
<6 months	16	21.0
6-12 months	5	6.6
1-3 years	12	15.8
>3 years	43	56.6
Location of gastrostomy insertion (n = 76)		
CHI at Crumlin	50	65.8
CHI at Temple St	17	22.4
CHI at Tallaght	9	11.8
Presence of NG/NJ tube prior to gastrostomy (n = 69)		
Yes	53	76.8
No	15	21.7
Not sure	1	1.5

Abbreviations: CHI, Children’s Health Ireland; NG, nasogastric; NJ, nasojejunal; IQR, interquartile range.

### Experience Prior to Gastrostomy Insertion

The most common reasons for the gastrostomy insertion (Table 2) were a child having an unsafe swallow (40.8%, n = 31) and a child experiencing significant oral feeding difficulties (n = 15, 19.7%). Caregivers rated their understanding of the reason for the gastrostomy tube insertion highly, with the median rating being 10 on a 10-point scale, indicating excellent understanding. The median satisfaction with the information provided by the multidisciplinary team prior to gastrostomy insertion was 10 on a 10-point scale (7.5-10.0), indicating excellent satisfaction. There was a statistically significant (*P *< .01) and moderate (rho* *= 0.385) positive correlation between ratings of understanding of the reason for the gastrostomy tube insertion and satisfaction with the information provided by the hospital team in advance of insertion.

**Table 2.** Caregiver Views on Aspects of the Gastrostomy Insertion Experience for their Child.

	Median	IQR
Rating of understanding of reason for gastrostomy insertion (n = 76)	10.0	10.0,10.0
Rating of satisfaction with information from hospital team (n = 72)	10.0	7.5,10.0
	n	%
Reason for gastrostomy insertion (n = 76)		
Unsafe swallow	31	40.8
Difficulty feeding your child	15	19.7
Poor weight gain	13	17.1
Poor nutrition/nutritional deficiency	5	6.6
For fluids only	1	1.3
For medications only	1	1.3
Other	10	13.2
Timing of gastrostomy tube insertion (n = 72)		
Inserted at the right time	52	72.2
Should have been inserted when my child was younger	20	27.8
Involvement in the decision-making process (n = 71)		
I always felt included	46	64.8
I sometimes felt included	14	19.7
I almost never felt included	6	8.5
Other	5	7.0

Abbreviation: IQR, interquartile range.

Most caregivers (72.2%, n = 52) felt that the gastrostomy tube was inserted at the right time (Table 2), while 27.8% felt that it should have been inserted when their child was younger. Almost two-thirds (64.8%, n = 46) of caregivers felt always included in the decision-making process.

### Experience of the Gastrostomy Insertion Period

Most caregivers (88.7%, n = 63) felt that they would make the same decision again to insert the gastrostomy tube (Table 3). Almost half (57.8%, n = 41) reported that their child does not feed orally and are fed solely via gastrostomy tube. Half (21.1%, n = 15) of the children with some oral intake (42.2%, n = 30) had not had any changes in their level of oral intake since the gastrostomy insertion.

**Table 3.** Caregiver Views of the Decision to Consent to a Gastrostomy Procedure for their Child After the Procedure.

	n	%
Current view of decision (n = 71)		
I would make same decision again	63	88.7
I would make a different decision	4	5.7
I don’t know what decision I would make	3	4.2
Other	1	1.4
Child's oral intake post insertion (n = 71)		
My child does not feed orally at all	41	57.8
My child's oral intake has not changed	15	21.1
My child's oral intake is better	10	14.1
My child's oral intake is worse	5	7.0
Spoke with another caregiver of a child with a gastrostomy tube prior to the procedure (n = 73)		
Yes	35	47.9
No	38	52.1

Almost half (47.9%) spoke with another caregiver of a child with a gastrostomy tube inserted, prior to the insertion procedure. Of those that did, 55% (n = 18) found it helpful to hear someone else's story and 33% (n = 11) felt more reassured about their child undergoing the procedure.

Following gastrostomy insertion, half (51.3%, n = 39) of respondents were concerned about how to manage the tube, 14.5% (n = 11) were concerned about how their child would feel about the tube, and 13.2% (n = 10) were concerned their child's appetite would decrease post-insertion. Other concerns included complications such as granulation tissue or infections, fear of the tube coming out, and that the gastrostomy was irreversible.

### Emotional Response to Gastrostomy Insertion Experience

**Figure 1.**
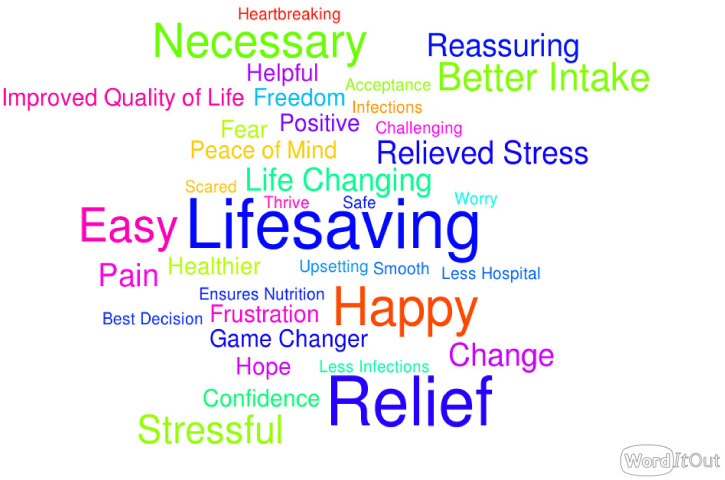
Caregiver descriptions of the gastrostomy tube insertion experience.

The most common words used by caregivers to describe their experience of gastrostomy tube insertion were “lifesaving,” “relief,” and “happy” (Figure 1). Of the 203 words and phrases submitted, 131 (64.5%) were categorized as positive, 25 (12.3%) as neutral, and 47 (23.1%) as negative. Common positive phrases included “improved quality of life,” “better intake,” and “best decision.” Common neutral phrases included “life changing,” “necessary,” and “acceptance.” Common negative phrases included “frustrating,” “stressful,” and “fear.”

The “other” answer box allowed caregivers to elaborate on their experience. Insights ranged from the positive impact of gastrostomy insertion, *“We felt very included in decision, looking back we should have done it sooner…felt like much bigger of a step than it turned out to be…” (ID70),* to less positive impacts, “*Upset…fear of tube falling out…feel like I’m on call all the time” (ID40)*, and neutral comments, such as “*A permanent change” (ID51).*

## Discussion

This was the first study in Ireland exploring the experiences of caregivers of children requiring a gastrostomy tube for some or all their nutritional needs. Key findings included the significant association between caregiver satisfaction and clarity from the multidisciplinary team on the need for gastrostomy insertion. Peer support from other caregivers was also identified as important during the transition to gastrostomy feeding, and common concerns following gastrostomy insertion highlighted the potential value of empowering caregivers to self-manage minor technical challenges following the insertion procedure.

Caregivers rated their satisfaction with the gastrostomy insertion highly, with caregivers who understood the reason for gastrostomy insertion being more satisfied with the information provided by the multidisciplinary team. A systematic review of parent experiences of gastrostomy identified that key issues for caregivers included dissatisfaction with the paucity of information on gastrostomy insertion, inconsistencies in the information provided between healthcare professionals (HCPs), and the unsuitable timing of information provision.^13^ It is possible that these findings were not evident in this study due to the progress made in recent years in ensuring a patient-centered approach to healthcare provision. This finding also highlights the benefit of accessible and timely education on caregiver satisfaction.^13^ Enhancing caregiver understanding of the reason for gastrostomy insertion through timely and comprehensive information provision may serve to address some of their stress and uncertainty. This may not always be possible (eg, in emergency decisions), but introducing the concept of gastrostomy feeding before it becomes an immediate reality for caregivers may assist them in understanding some of its fundamental principles before making the decision on whether their child should undergo gastrostomy insertion.

Almost half of caregivers spoke to another caregiver of a child with a gastrostomy tube inserted, prior to the insertion procedure. It is notable that of those who did, the overwhelming majority reported benefits from this source of support. Although this study highlights the potential significant benefits of caregivers communicating with others in a similar position to them, over half of caregivers in this study did not have the opportunity engage with other caregivers before gastrostomy insertion. Parents in other studies have expressed the desire to communicate with others in a similar position to them, stating that they would be able to empathize with them and support each other, due to another caregiver's lived experience of gastrostomy feeding.^12^ Additionally, peer support networks for parents of children with a neurodisability have also been shown to enhance the resilience of not just the caregiver, but the entire family.^17^ There is a lack of research exploring the impact of peer support between caregivers during gastrostomy insertion, making this an area that may benefit from further investigation.

The most common concern that caregivers had following the insertion of the gastrostomy tube related to its management, followed by how a child felt about the tube, and a child having a reduced appetite because of the tube. This information can inform HCPs on the issues that caregivers consider most worrying and facilitate conversation on strategies that address concerns in a timely manner. It is worth noting that some of these common concerns are those that caregivers can be empowered to self-manage, given the correct skills and resources.^19^ Access to user-friendly instructional videos or guides may provide caregivers with information and support at the time that the specific concern arises.^20,21^ These resources should be evidence-based and allow for input from HCPs and caregivers. The aim would be to empower caregivers with the skills to deal with minor issues as they arise. HCPs remain fundamental to the process of tube management, but equipping caregivers with the skills to independently manage some of these concerns is a worthwhile endeavor to improve the day-to-day care of service users.^22^

It is notable that the oral intake of most of the children in this study who had some oral intake prior to gastrostomy insertion, either did not change or increased since the gastrostomy insertion. This reflects positively on the healthcare these children received to maintain their oral skills and nutrition, even with the gastrostomy tube in place, and may serve to reassure caregivers who are concerned about reductions in their child's oral intake.

The limitations of this study are acknowledged. Although this study was conducted in the largest pediatric acute care setting in Ireland and has a similar sample size to other studies exploring this topic,^13^ the sample size is modest and so larger, nationally representative studies are warranted. There was no single database available to determine the exact number of children under the care of CHI that met the eligibility criteria for this study, so the proportion of the population represented in this study can only be estimated. Based on dietetic records across the 3 CHI hospitals, approximately 460 children were receiving HEN under the care of CHI at the time of the study, so about 16% of the eligible population may be represented in this study. While the survey had an 87% completion rate, convenience sampling was used and participants were self-selected, so parents with more positive or more negative experiences may have been more likely to participate in the study. The study design was cross-sectional and retrospective, with most children being well-established with their feeding tubes, so recall bias must be considered. Time constraints during the data collection period meant that it was not possible to validate the survey beyond face validity. Validated instruments were not available to answer the research question, and so this is an area for potential future research. Additionally, this sample was not stratified by age of the child at gastrostomy insertion, so larger studies that can facilitate this level of analysis would be of benefit, as caregiver experiences of this process are likely to differ between younger and older children. Additionally, most caregivers in this study were mothers, and so capturing the perspectives of fathers and other caregivers in future research would also be of benefit. Further research on the impact of introducing the concept of gastrostomy feeding to caregivers at an earlier stage would also be beneficial.

The decision to commence gastrostomy tube feeding remains a complex one for parents and caregivers, as it has considerable implications for themselves, their child, and their family.^13,14^ This study demonstrates the value of sufficient and timely education and the fostering of a strong caregiver–HCP relationship. It highlights the importance of support between caregivers, and of empowering caregivers with the skills, resources, and confidence to manage minor complications independently. Multidisciplinary teams in pediatric settings could consider the development of formal care pathways that aid caregiver decision-making, skill development, and access to peer support at appropriate intervals to ensure the best possible outcomes for each child and family undergoing the transition to this form of nutritional support.
